# Association of self-regulation with white matter correlates in boys with and without autism spectrum disorder

**DOI:** 10.1038/s41598-020-70836-y

**Published:** 2020-08-14

**Authors:** Hsing-Chang Ni, Hsiang-Yuan Lin, Wen-Yih Isaac Tseng, Susan Shur-Fen Gau

**Affiliations:** 1 Department of Psychiatry, Chang Gung Memorial Hospital, Linkou, Taiwan; 2 Graduate Institute of Clinical Medicine, National Taiwan University College of Medicine, Taipei, Taiwan; 3 Department of Psychiatry, National Taiwan University Hospital and College of Medicine, Taipei, Taiwan; 4 Azrieli Adult Neurodevelopmental Centre, Centre for Addiction and Mental Health, Toronto, ON Canada; 5 Department of Psychiatry, University of Toronto, Toronto, ON Canada; 6 Graduate Institute of Brain and Mind Sciences, College of Medicine, National Taiwan University, Taipei, Taiwan; 7 Institute of Medical Device and Imaging, College of Medicine, National Taiwan University, Taipei, Taiwan

**Keywords:** Neuroscience, Emotion, Neural circuits, Social neuroscience

## Abstract

Previous studies demonstrated distinct neural correlates underpinning impaired self-regulation (dysregulation) between individuals with autism spectrum disorder (ASD) and typically developing controls (TDC). However, the impacts of dysregulation on white matter (WM) microstructural property in ASD and TDC remain unclear. Diffusion spectrum imaging was acquired in 59 ASD and 62 TDC boys. We investigated the relationship between participants’ dysregulation levels and microstructural property of 76 WM tracts in a multivariate analysis (canonical correlation analysis), across diagnostic groups. A single mode of brain-behavior co-variation was identified: participants were spread along a single axis linking diagnosis, dysregulation, diagnosis-by-dysregulation interaction, and intelligence to a specific WM property pattern. This mode corresponds to diagnosis-distinct correlates underpinning dysregulation, which showed higher generalized fractional anisotropy (GFA) associated with less dysregulation in ASD but greater dysregulation in TDC, in the tracts connecting limbic and emotion regulation systems. Moreover, higher GFA of the tracts implicated in memory, attention, sensorimotor processing, and perception associated with less dysregulation in TDC but worse dysregulation in ASD. No shared WM correlates of dysregulation between ASD and TDC were identified. Corresponding to previous studies, we demonstrated that ASD and TDC have broad distinct white matter microstructural property underpinning self-regulation.

## Introduction

Autism spectrum disorder (ASD) is a neurodevelopmental disorder that encompasses the impairments in social interaction and communication and restricted, repetitive patterns of behaviors^1^. Besides the core symptoms, impaired self-regulation (namely dysregulation) in ASD is also common^2,3^. Generally, individuals’ optimal self-regulation can facilitate flexible modification of their interoceptive state and modulation of response to exteroceptive stimuli^4^. Suboptimal self-regulation is associated with heightened risk for affective psychopathology^5^ and maladaptive behaviors^6–8^. Dysregulation in ASD also links to higher use of psychiatric services, more social impairment, lower family quality of life, and more depressive and anxiety symptoms^9–13^. Dysregulation associated with ASD may be explained by co-occurring psychiatric disorders, maladaptive strategies, less-frequent use of cognitive reappraisal, and inherent autistic psychopathology^2,14,15^.

Self-regulation is a theoretically complex construct, which involves the affective, behavioral, and cognitive control^16^. Specifically, emotion regulation involves dual processes, which initiate regulation contingent on explicit and implicit goals as supported by controlled and automatic processes, repsectively^17^. Cognitive control interacts with motivation and may be treated as a domain of reward-based decision making^18^. Behavioral regulation is theoretically associated with belief salience measures, past behavior/habit, perceived behavioral control, self-efficacy, moral norms, self-identify, and affective beliefs^19^. However, such essential elements of self-regulation tend to work synergistically and show ongoing and dynamic modulation of each other^16^. Correspondingly and similarly, neural correlates of affect regulation implicate the amygdala, insula, ventromedial prefrontal (vmPFC)/orbitofrontal (OFC) cortex, ventrolateral (vlPFC), dorsolateral prefrontal cortex (dlPFC), and anterior cingulate cortex^17^. These prefrontal and cingulate components of affect regulation encompass the key regions involved in cognitive^18^ and behavioral control^20^. Of note, different striatal and subcortical regions may distinctly contribute to affect^21^, cognitive^22^, and behavioral control^23^.

To sum up, affect, behavioral, and cognitive control teams up with each other to facilitate self-regulation. Following this collective perspective, the level of dysregulation could potentially be measured by the Child Behavioral Checklist-Dysregulation Profile (CBCL-DP)^24^, which characterizes co-occurring elevations on the Anxiety-Depression (affect), Aggression (behavior), Attention (cognition) subscales on the CBCL in both clinical^24^ and non-clinical populations^25^. Previous study demonstrated that the CBCL-DP is different from CBCL total scores and specific for distinct adult outcomes^26^. The CBCL-DP profile has been shown to estimate the deficit emotional self-regulation and predict the functional impairment across several psychiatric disorders^27^. We also have applied this CBCL-DP to investigate structural^28,29^ and intrinsic functional correlates^30^ of dysregulation associated with ASD.

Despite the increasing understanding of the importance of self-regulation in ASD, the neural correlates underlying self-regulation in ASD are far from conclusive^31^. For example, using functional magnetic resonance image (fMRI), Richey et al.^32^ found children with ASD, relative to TDC, have less increase in the nucleus accumbens and amygdala and less change in the dlPFC activation during cognitive reappraisal of faces. Pitskel et al.^33^ found TDC show downregulation of bilateral insula and left amygdala while children with ASD show no modulation of insula and upregulation of the left amygdala on the decrease trials of emotional responses to disgusting images. Based on the CBCL-DP, our recent resting-state fMRI study demonstrated that ASD and TDC have distinct intrinsic functional connectivity in relation to dysregulation^30^. Overall, these fMRI studies have demonstrated that ASD and TDC appear to have different regional brain activities and connectivity in processing self-regulation. However, investigations based on structural MRI suggest the hypothesis asserting distinct neural mechanisms underpinning self-regulation in ASD and TDC remains contentious^28,29^.

As the development of white matter (WM) connectivity speaks to coordinated gray matter growth and functional network organization, WM tracts interconnecting the preceding regions may also distinctly relate with dysregulation in ASD. Diffusion-weighted MRI (dMRI) is a powerful method to characterize the organization and architecture of WM fibers^34^, by estimating the water diffusion profile in the brain. Although prior reports have applied dMRI to portray diagnosis-distinct WM microstructural properties in several psychiatric disorders with impaired self-regulation, such as major depressive disorder^35^, and bipolar disorders^36^, only one study has explored dimensional WM correlates of self-regulation in healthy adults^37^. Vandekerckhove et al.^37^ demonstrated that groups using high and low bottom-up emotional regulation have different WM microstructural properties in the tracts supporting emotion regulation, cognitive and motor control, and sensory affective processing information. Earlier literature suggests that the most consistent alterations of WM microstructural properties and organization in ASD involve corpus callosum and superior longitudinal fasciculus^38,39^, which might also be implicated in self-regulation given their respective roles in whole-brain information integration (corpus callosum) and communication within frontoparietal and frontotemporal systems (superior longitudinal fasciculus)^40^. Nonetheless, to our best knowledge, no study has yet investigated WM correlates underpinning dysregulation in ASD, which hampers a better understanding of this critical problem in ASD.

To investigate WM correlates of dysregulation in intellectually able boys with ASD and TDC boys, we leveraged recent advances in multivariate analysis, canonical correlation analysis (CCA)^41^, and the diffusion spectrum imaging (DSI) tractography^42^. DSI, relative to popular diffusion tensor imaging (DTI), was intentionally adopted to enable the detection of crossing WM fiber bundles, which have pronounced effects on tractography^43^. We hypothesized that the ASD and TDC groups would have distinct associations of dysregulation with microstructural property in widespread WM tracts, which interconnect regions involved in cognitive, affective, and behavioral controls.

## Results

The ASD and TDC groups in the main sample (59 ASD and 62 TDC) had comparable demographic features, including age, handedness, intelligence, and in-scanner head motion levels (signal dropout counts) and DSI data signal-to-noise ratio (SNR) (Table 1). The psychiatric comorbidity and concurrent methylphenidate use is shown in Supplementary Table 1.

**Table 1:** Demographic and clinical features of the main sample.

Mean (SD)	ASD (n = 59)	TDC (n = 62)	Statistics *P* value
**Age** (years)	12.5 (1.7)	12.0 (2.1)	0.199
**Handedness**, right (%)	55 (91.7)	60 (96.8)	0.432
**Full-scale IQ**	106.6 (14.3)	110.2 (11.3)	0.129
Verbal IQ	107.1 (14.1)	110.1 (10.9)	0.214
Performance IQ	105.1 (16.2)	109.1 (13.1)	0.150
**Impaired self-regulation**	204.8 (42.9)	151.0 (30.8)	< 0.001
**Autism diagnostic interview-revised**^a^			
Social	9.7 (5.3)	–	
Communication	8.8 (4.1)	–	
Repetitive and stereotyped behaviors	5.1 (2.7)	–	
**Head motion and image quality**			
Signal-to-noise ratio	27.9 (2.9)	27.3 (2.6)	0.217
Signal dropout counts	13.7 (15.6)	10.8 (12.5)	0.259

^a^Based on the current behavior algorithms.

ASD, autism spectrum disorder; TDC, typically developing controls; IQ, intelligence quotient; SD, standard deviation.

Seventy-six white matter tracts of the whole brain were identified by the tract-based automatic analysis (TBAA) method^42^, and their generalized fractional anisotropy (GFA) values were calculated^44^. After controlling for age linear and squared terms, signal dropout counts, and SNR, the diagnostic differences in WM property did not survive correction for multiple tests by false discovery rate, FDR^45^ (Supplementary Table 2).

**Table 2:** Similar and different associations between dysregulation and GFA values between autism spectrum disorder (ASD) and typically developing controls (TDC) based on the main sample.

Pattern	Tract	Connected ROIs	Connected ROIs	System
**Similar association**	Not significant			
**Different association**				
(a) The lower GFA values with the worse regulation in ASD/the better regulation in TDC				
	Left ILF	L_temporal pole	Occipital lobe	Emotion recognition and visual-affective integration
	Left UF	L_orbitofrontal gyrus	L_superior temporal pole	Emotion regulation
	CC of genu	L_frontal components, including orbitofrontal gyrus	R_frontal components, including orbitofrontal gyrus	high cortical function regulation
(b) The higher GFA values with the worse regulation in ASD/the better regulation in TDC				
	L_CST of mouth	L_primary motor cortex of mouth component	Brain stem	Motor
	L_CST of toe	L_primary motor cortex of toe component	Brain stem	Motor
	L_CST of geniculate fibers	L_primary motor cortex of throat component	Brain stem	Motor
	L_CST of trunk	L_primary motor cortex of trunk component	Brain stem	Motor
	L_CST of hand	L_primary motor cortex of hand component	Brain stem	Motor
	R_CST of geniculate fibers	R_primary motor cortex of throat component	Brain stem	Motor
	R_CST of trunk	R_primary motor cortex of trunk component	Brain stem	Motor
	R_CST of toe	R_primary motor cortex of toe component	Brain stem	Motor
	L_TR of auditory nerve	L_thalamus	L_Heschl’s gyrus	Sensory processing
	L_TR of precentral gyrus	L_thalamus	L_precentral gyrus	Sensorimotor integration
	R_TR of postcentral gyrus	R_thalamus	R_postcentral gyrus	Sensory processing
	L_cingulum of hippocampal component	L_cingulate gyrus posterior part	L_hippocampus	Emotion, memory
	L_cingulum of the main body component	L_cingulate gyrus (anterior + middle part)	L_cingulate gyrus posterior part	Attention, emotion, cognitive control
	Posterior commissure	Dorsal aspect of the upper end of the cerebral aqueduct	Bilateral cerebral hemispheres	Pupillary light reflex and upward saccade, both related to automatic emotion perception
	R_frontal aslant tract	R_SMA	R_inferior frontal gyrus opercular part	Inhibition
	L_medial lemniscus	L_thalamus	Brain stem	Somatosensory

CC, corpus callosum; CST, corticospinal tract; ILF, inferior longitudinal fasciculus; L, left; R, right; SMA, supplementary motor area; TR, thalamic radiation; UF, uncinate fasciculus.

**Figure 1:**
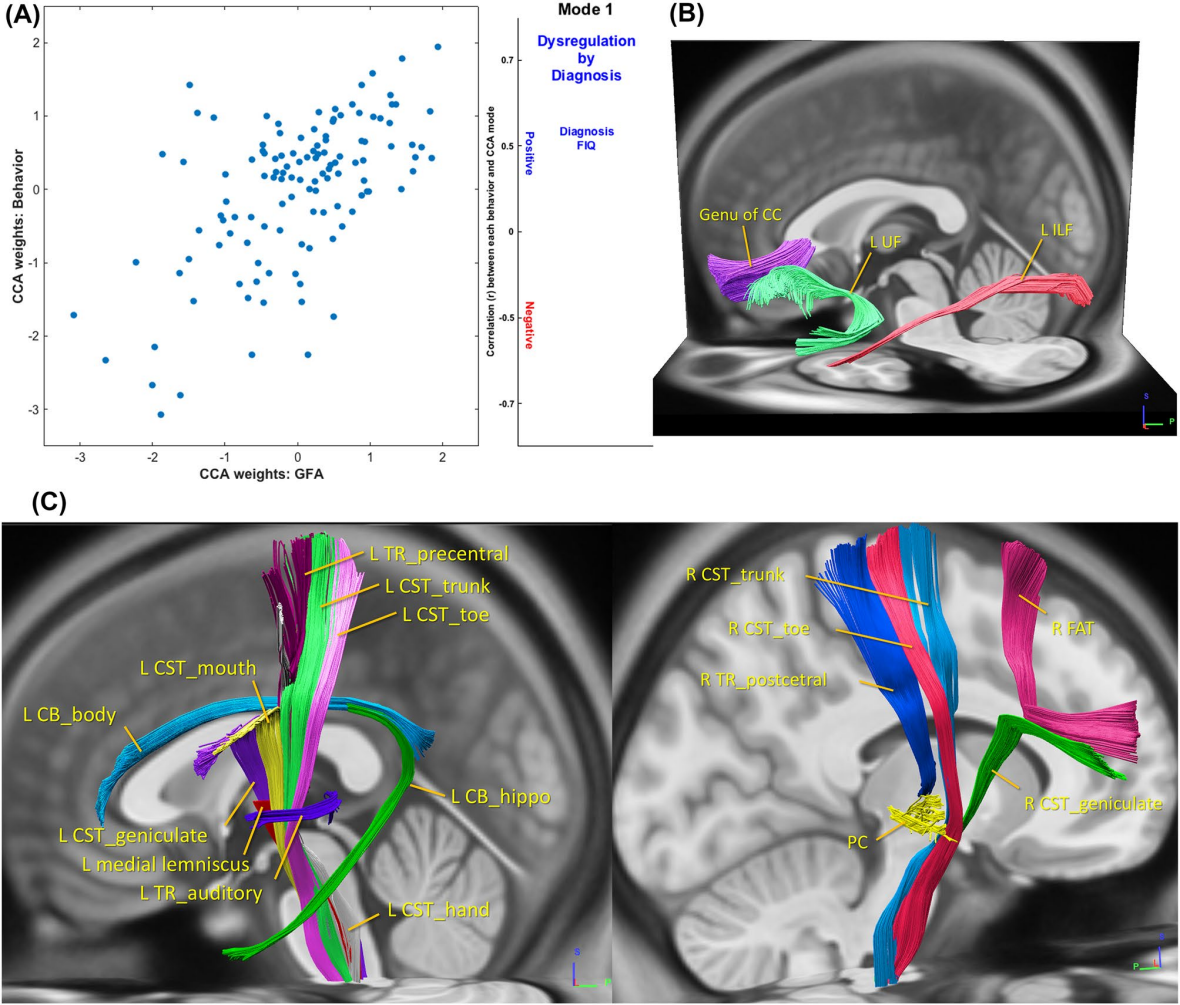
Canonical correlation analysis (CCA) mode relating microstructural property of 76 white matter tracts to dysregulation levels and cognitive measures across the whole cohort. (**A**) The CCA analysis identified a single significant (FWE-corrected *p* = 0.005) mode of associations between white matter microstructural property and the behavioral variables of interest. The strength and direction of the variance explained by the CCA mode are indicated in the figure by the vertical position and font size. (**B**) Higher dysregulation levels in ASD, while lower dysregulation levels in TDC, were negatively correlated with white matter property of a set of 3 tracts including the left uncinate fasciculus (UF), left inferior longitudinal fasciculus (ILF) and genu of corpus callosum (CC). (**C**) Higher dysregulation levels in ASD, while lower dysregulation levels in TDC, were positively correlated with white matter property of a set of 16 tracts including the main body and hippocampal components of the left cingulum (CG), hand and mouth part of the left corticospinal tract (CST), toe, trunk and throat part of bilateral corticospinal tracts, left thalamic radiation (TR) linking to the precentral gyrus and auditory nerve, right thalamic radiation linking to the postcentral gyrus, right frontal aslant tract, left medial lemniscus and the posterior commissure, left thalamic radiation linking to the precentral gyrus and auditory region, right thalamic radiation linking to the postcentral gyrus, right frontal aslant tract (FAT), left medial lemniscus and the posterior commissure (PC). The tracts depicted here are the tractogram reconstructed in the DSI template, which derived from the average of diffusion datasets of 122 healthy adults^42^. The exact delineation of the tracts varied somewhat between individuals (see Supplementary Fig. 2). R, right; L, left; GFA, generalized fractional anisotropy.

CCA applies a multivariate approach to identify latent linear brain-behavior relationships^41^ between sets of independent (behavioral measures) and dependent (brain measures) variables^46^. The first mode of CCA estimates the maximum co-variation between these two sets of the brain and behavioral measures. The maximum residual, orthogonal co-variation is represented by subsequent modes. One significant mode (*r* = 0.59, FWE-corrected *p* = 0.005) of interdependencies between WM property patterns and the diagnosis, diagnosis by dysregulation interaction and general cognitive function (full-scale intelligence quotient, FIQ) (Fig. 1A and Supplementary Table 3) was identified by CCA. Specifically, lower GFA values of 3 tracts, including the left uncinate fasciculus, left inferior longitudinal fasciculus (ILF) and genu of the corpus callosum, were positively associated with higher dysregulation in ASD, but lower dysregulation levels in TDC (Fig. 1B, Table 2 and Supplementary Table 4). FIQ and diagnosis (as expressed as ASD > TDC) were also negatively associated with WM property patterns of this set of tract bundles. The directions of the diagnostic differences which drove this set of WM tracts identified in the first CCA mode were compatible with that found in the conventional generalized linear model (Supplementary Table 2).

On the other hand, higher GFA values of 16 tracts were positively associated with higher dysregulation in ASD but lower dysregulation levels in TDC (Fig. 1C, Table 2, and Supplementary Table 4). These 16 tracts included the main body and hippocampal components of the left cingulum, the hand and mouth part of the left corticospinal tract, the toe, trunk and throat part of bilateral corticospinal tracts, the left thalamic radiation linking to the precentral gyrus and auditory nerve, the right thalamic radiation linking to the postcentral gyrus, the right frontal aslant tract, the left medial lemniscus, and the posterior commissure, the left thalamic radiation linking to the precentral gyrus and auditory region, the right thalamic radiation linking to the postcentral gyrus, the right frontal aslant tract, the left medial lemniscus and the posterior commissure. The other 3 modes did not capture any additional meaningful association between patterns of WM property and behaviors (*p* = 0.080, 0.229, 0.479, respectively).

To test the robustness of our results, we adopted two strategies. First, we implemented a “leave-five-out per diagnostic group” sensitivity analysis with 1,000 permutations. The average *p* value of the first CCA mode was 0.015, and 95.3% of *p* values among these 1,000 permuted CCA was smaller than 0.05. Besides, we implemented the same CCA in the originally recruited sample (87 ASD and 77 TDC), which exhibited significant between-group differences in age and intelligence (Supplementary Table 5). This additional CCA also identified one significant mode (*r* = 0.61, FWE-corrected *p* = 0.032; Supplementary Table 6) of interdependences between WM property patterns and diagnosis by dysregulation interaction and FIQ. Similarly distinct pattern of WM property and dysregulation in ASD and TDC was also found in this larger originally-recruited sample (Supplementary Fig. 1). The set of WM bundle tracts as identified in this additional CCA (Supplementary Table 7) overlapped with some of those from the initial results (8 out of 19), but showed largely consistent patterns in the involved functional systems (Supplementary Table 8).

## Discussion

The current study is the first to investigate the neural correlates underpinning self-regulation in ASD and TDC based on WM microstructural property. By using the state-of-the-art DSI tractography and multivariate approach, we observed that ASD and TDC had distinct associations of self-regulation with microstructural property in diverse WM tracts, which may further contribute to altered strategies and the presentations of emotion regulation and cognitive control in ASD.

We identified that lower GFA values were associated with higher dysregulation in ASD but lower dysregulation in TDC in the left ILF, left uncinate fasciculus, and genu of the corpus callosum. The ILF interconnects the anterior temporal pole (affective) and occipital lobe (visual) and is involved in face recognition, semantic processing, visual memory, and emotion^47^. Earlier studies demonstrated the strong association of ILF and recognition of facial emotions^48,49^. Lesions corresponding to the ILF are associated with impaired processing of affective facial expressions^50^. The uncinate fasciculus is an association tract that connects the amygdala and hippocampus with the orbitofrontal cortex and supports emotional empathy^51^ in patients with hemispheric stroke, and emotion regulation^52^ in neurotypical adults. Moreover, the microstructural integrity of the uncinate fasciculus is significantly correlated with the performance on the emotionally reading the mind in the Eyes Task in neurotypical adults^53^ and can identify the at-risk group for bipolar disorder and the group with non-bipolar psychopathology (i.e., depression and ADHD)^54^. The genu of the corpus callosum is a commissure tract connecting the bilateral orbitofrontal cortex, which is critically implicated in emotion regulation^55^, as well as connecting medial and lateral surface of the frontal regions. Previous studies demonstrated that the microstructural integrity of the genu of CC correlated with suicidal behaviors in bipolar disorder^56^ and several emotional dysregulation associated psychiatric disorders including borderline personality disorder^57^, major depressive disorder^58^, bipolar disorder^59^, and post-traumatic stress disorder^60^.

Jointly, the WM tracts mentioned above interconnecting prefrontal components, the OFC and limbic, and visual and affective systems, are critical in the self-regulation of socio-emotional behaviors^31^. The result that higher GFA values of this set of circuitries were associated with a dysregulation in TDC is largely consistent with WM^37^, functional^17^ and morphometric correlates^61^ of self-regulation, as reported in earlier literature on neurotypical adults. Notably, the direction of this brain-behavior relationship reversed in the ASD group (diagnosis by dysregulation interaction), suggesting that ASD and TDC have different patterns of associations between dysregulation and white matter microstructural property^32,33,62,63^. Our results endorse the hypothesis that alterations in circuitries linking OFC and limbic systems contribute to dysregulatory socioemotional behaviors in ASD^31^.

Simultaneously identified in this significant CCA mode, higher GFA was associated with higher dysregulation in ASD but lower dysregulation in TDC in several major fiber traits, as described below. The central part of the cingulum bundle (dorsal cingulum) is implicated in attention^64,65^ and executive control, and emotion. The hippocampal part of the cingulum (ventral or parahippocampal cingulum) is closely linked to the learning, episodic memory^66^, and manage emotion^67^. The corticospinal tract, connecting the brain stem and primary motor cortex, mainly supports motor processing and voluntary movement^68^. The subdivisions of these corticospinal tracts are based on previous reports^68,69^. Interestingly, the microstructural property of the corticospinal tract is also suggested to be associated with understanding emotions^67^. The thalamic radiations of precentral and postcentral parts connect the thalamus with precentral and postcentral gyrus and are critical relays within the motor and somatosensory network^70,71^. The left medial lemniscus connecting the thalamus and brain stem is associated with motor planning and execution^72^. The right frontal aslant tract connecting the supplementary motor area and inferior frontal gyrus is associated with executive function especially inhibitory control^73^. The posterior commissure is a commissural connection between the cerebrums and is suggested to be implicated in upward saccade and pupillary responses, which are both related to automatic emotion perception^74^. Altogether, these 16 tracts work in parallel to support top-down regulation, such as attention and inhibition, as well as bottom-up processes including memory, sensory-motor integration, motor processing, and automatic perception. Beyond the facets related to emotion, the process of self-regulation intricately implicates in several neuropsychological constructs such as executive and attention control, response inhibition, motor planning, and emotion reactivity and regulation^16^. The coordinated interplay between top-down and bottom-up systems is suggested to support many cognitive functions included under the ‘self-regulation’ umbrella, such as attention^75^, perception^76^, behavioral inhibition^77^, and emotion generation^78^. Together with the aforementioned results of tracts connecting OFC, visual, and limbic systems, our findings echo an earlier hypothesis conjecturing that emotion dysregulation in ASD might be related to cognitive regulation, informative processing, perception, or altered physiological activity^2^.

Our speculation that dysregulation in ASD is contributed by a complex interplay between top-down and bottom-up systems was further supported by the sensitivity analysis based on the originally recruited sample. Despite potential confounding effects from between-group age, intelligence, and DSI SNR differences in this larger cohort, around half of the identified tracts in this additional CCA overlapped with that set using the main sample. Those seemingly inconsistent tracts identified in the additional CCA were actually also involved in the foregoing functional systems (Supplementary Table 8). For example, the left superior longitudinal fasciculus I was identified in the WM set from additional CCA, and is involved in regulating motor behaviors and voluntary oriented visuospatial attention^79^. The left perpendicular fasciculus was a similar case, given its critical role in attention and eye movement control^80^. These concordant findings also indirectly endorse the validity of using the CBCL-Dysregulation, which could exhaustively capture the miscellaneous features of the dysregulation, in the present study.

Besides the findings of distinct WM correlates underpinning self-regulation, we also observed interdependence between FIQ as well as diagnosis, and the aforementioned WM property patterns in the significant CCA mode. This result provides evidence to endorse the notion that the level of intelligence may be linked to alterations in neural circuitries that are involved in self-regulation in ASD^31^. The direction of diagnostic differences in this set of 19 tracts, which partly contributed to the first CCA mode, was compatible with the results from the conventional generalized linear model (Supplementary Table 2). However, these autism-associated WM alterations based on the univariate analysis did not survive correction for multiple tests, which are incompatible with the previous studies^81^. The reasons for this lack of diagnostic differences may be related to some issues. A major source of inconsistency is certainly the fact that sample characteristics^82^, especially sex effects^83^, are confounded.

On the one hand, our male-only sample may reduce sex-related heterogeneity; on the other hand, this approach may limit comparability with other studies. Methodologically, the present study applied the template-based whole-brain tractography based on DSI data (TBAA) to investigate microstructural WM properties, particularly GFA^42^. Despite its methodological sensitivity and specificity, the current findings should be complemented by other dMRI data, such as high angular resolution diffusion imaging and DTI, other template-based tract-specific analysis, e.g., tract-based spatial statistics^84^, and another approach estimating structural connectivity^85^. Moreover, despite a high correlation between GFA and fractional anisotropy^86,87^, we should refrain from directly comparing the findings from DSI and DTI without considering their different biophysical models and assumptions^88^. In addition, the present null diagnostic difference based on the univariate analysis may reflect our emphasis on comparable levels of head motion between ASD and TDC, as emerging evidence has indicated that in-scanner motion tends to produce spurious group differences^89^.

Some other limitations need to be mentioned. First, the current intellectually able sample of ASD may limit the generalization of the present findings to others on the spectrum^90^. In addition, considering common co-occurrence, we included children with ASD co-occurring with ADHD in the study. The ratio of psychiatric comorbidity in our ASD group (Supplementary Table 1) is largely compatible with the previous literature^91^. Nonetheless, this caveat regarding inclusion criteria may influence the present results, as ADHD is also highly associated with impaired self-regulation^92,93^. Furthermore, the concurrent use of methylphenidate might also have effects on the WM microstructure^94^. Future studies, including individuals with a broader functional presentation on the autism spectrum as well as individuals with ADHD-only, could help address this limitation. Second, we only used the CBCL to estimate dysregulation levels. Despite the validity of CBCL-Dysregulation^24^, this profile could only capture proxy dysregulation information cardinally containing affective, behavior, and cognitive domains. Other approaches, such as emotion dysregulation inventory^95^, could be adopted in the future study.

Our results provide the first evidence that ASD and TDC have distinct associations of dysregulation with the properties of WM tracts involved in emotion, attention control, sensorimotor processing, and visual-affective integration. Our results should be considered robust based on the consistent findings from two different strategies of sensitivity analyses. These findings not only support a notion that self-regulation encompasses multiple cognitive processes, but also suggest indispensable diagnosis-specific strategies when advancing therapeutics for dysregulation in individuals with ASD.

## Methods and materials

### Participants and procedures

We restricted the recruitment to males because of differences in white matter properties between ASD girls and boys, and also a relatively low prevalence of female patients with ASD^96,97^. Study participants hence included 104 Taiwanese boys with ASD from the child psychiatric outpatient clinic of National Taiwan University Hospital (NTUH) and 90 TDC boys from similar geographical areas. Structural MRI (T1-weighted) data of some participants have been published elsewhere^28^. The diagnosis of ASD was first assessed by the corresponding author (SSG) based on the DSM-IV-TR and ICD-10 criteria and further confirmed by interviewing the parents with the Chinese version of the Autism Diagnostic Interview-Revised (ADI-R). The age range of participants was 7–17 years, and their FIQ was higher than 70. The intellectual function was assessed Wechsler Intelligence Scale for Children–3rd Edition (for those younger than 16 years) and Wechsler Adult Intelligence Scale-3rd Edition (for the remaining older participants).

To confirm the psychiatric comorbid conditions, all the participants accepted the assessment of the Chinese version of the Schedule for Affective Disorders and Schizophrenia for School-Age Children–Epidemiological Version (K-SADS-E) interview^98–100^. The exclusion criteria for ASD and TDC were different. For ASD, we excluded the lifetime and current major psychiatric disorders such as schizophrenia, mood disorder, and substance use disorders, while autistic participants with the lifetime or current co-occurring attention-deficit/hyperactivity disorder, oppositional defiant disorder, learning disorder, and tic disorder were included in our study. To decrease the impact of anxiety on our results, the lifetime co-occurring anxiety disorder was included, but current co-occurring anxiety disorder was excluded. For TDC, we excluded any lifetime and current DSM-IV psychiatric disorders such as schizophrenia, mood disorder, anxiety disorder, substance use disorders, ADHD, learning disorder, and tic disorder. Participants with current or lifetime severe medical or neurological illness (e.g., epilepsy) and psychotropic medication, except methylphenidate, were also excluded from the study. The Research Ethics Committee at NTUH approved our study before implementation (200903062R, 201201006RIB; ClinicalTrials.gov number, NCT00916851, NCT01582256). Besides the ethical standards of the Committee at the NTUH on human experimentation, all procedures contributing to this work also comply with the Helsinki Declaration of 1975, as revised in 2008. Written informed consent forms were obtained from the participants and their parents after the detailed face-to-face explanations of the current study objectives and procedures.

### Assessments of dysregulation by the Child Behavior Checklist (CBCL)

The CBCL is a parent-reported scale to evaluate the behavioral problems of children aged 4–18. Among 118 items, eight subscales were categorized including attention, anxiety/depression, aggression, delinquency, social problems, somatic symptoms, thought problems, and withdrawn^101^. Raw scores of each subscale were transformed to *T*-scores with a mean of 50 and a standard deviation of 10 based on a norm of typically developing children and adolescents. The level of dysregulation was assessed by the sum of the T-scores from 3 subscales including Attention, Aggression, and Anxiety-Depression, as defined and used in the previous studies^24,102^.

### Image acquisition

This study adopted DSI, rather than DTI, for its better capacity to deal with the issues of crossing fibers and to unravel complex structural information^103^.

T1-weighted images and DSI were acquired on a 3 T MRI system (Siemens Magnetom Tim Trio, Germany) using a 32-channel phased arrayed head coil. High-resolution T1-weight imaging was performed using a 3D magnetization prepared rapid acquisition gradient echo sequence: Repetition time (TR)/Echo time (TE) = 2,000/2.98 ms; Inversion time = 900 ms; flip angle = 9°; field of view = 256 mm × 192 mm; matrix size = 256 × 192 × 208; voxel size = 1 mm^3^. DSI was performed using a pulsed-gradient spin-echo echo planner imaging with a twice-refocused balanced echo sequence^104^. The DSI sequence comprised 102 diffusion-encoding directions corresponding to the grid points within a half-sphere of the 3D diffusion encoding space (q-space) with the maximum diffusion sensitivity value (b_max_) of 4,000 s/mm^2^^105^. The grid points had coordinates with equidistance of 1 unit, and the coordinates of the grid points (qx, qy, qz) satisfied the relationship: (qx^2^ + qy^2^ + qz^2^) ≤ r^2^, where r was the radius specified by the DSI scheme. To reduce the scan time, the grid points contained within the half-sphere with radius r were acquired. The grid points outside the half-sphere were filled with zeros. Moreover, owing to the symmetric property of q space signal about the origin, only half of the q space data in the + z direction were acquired. For DSI with diffusion data acquired at 102 grid points within the half q space, the radius r was set at 3.6. The b values at grid coordinates were scaled according to the corresponding r values, referenced to bmax = 4,000 s/mm^2^ at r = 3. The other parameters were: TR/TE = 9,600/130 ms, FOV = 200 mm × 200 mm, acquisition matrix = 80 × 80, in-plane spatial resolution = 2.5 × 2.5 mm, slice thickness = 2.5 mm, and slice number = 54.

For the control of head motion, all participants were requested to lie still on the table with head movement restricted by expandable foam cushions. Besides, the DSI data underwent a quality assurance procedure to ensure acceptable in-scanner head motion by counting the number of diffusion-weighted images with signal dropout in the DSI datasets^89^. DSI datasets with more than 90 signal dropout images were discarded^42^. The DSI images of each individual (5,508 images per person, 102 (directions / slice) × 54 (slices / head)) were scrutinized by calculating the signals in the central square (20 × 20 pixels) of each image. Signal dropout was defined as the average signal intensity of an image lower than two standard deviations from the mean of all images (after correcting for its b value)^42^. Besides, we calculated the SNR based on the signal statistics in two predefined regions of interest, one placed in the central slice of the brain and the other in the background^106^. DSI datasets with SNR lower than 20 were excluded from the analysis.

Seventeen ASD and 13 TDC were excluded from further imaging processing owing to excessive in-scanner head motion and low SNR, yielding a sample of 87 boys with ASD and 77 TDC boys (the henceforth ‘originally recruited sample’). Our preliminary analysis found significant differences in age and intelligence between ASD and TDC. Such differences may strongly confound the findings. Therefore, we further matched the age and intelligence between the two groups, yielding a final sample of 59 boys with ASD and 62 TDC boys (the ‘main sample’ henceforth) for the initial data analysis. The main results were analyzed based on this main sample of 59 ASD and 62 TDC. We ran a sensitivity analysis by implementing an additional CCA based on the originally recruited sample (87 ASD and 77 TDC). These additional results are shown in Supplementary Tables 5–8 and Supplementary Fig. 1.

### DSI image reconstruction

The DSI data were reconstructed based on the Fourier relationship between the probability density function (PDF) and q-space signal^107^. Three-dimensional Fourier transform was performed on the q-space signal, applied with a Hanning filter of 17 units in width, to reconstruct the PDF. The orientation distribution function (ODF) was determined by computing the second moment of the PDF along each of the 362 radial directions in a sixfold tessellation^108^. Herein, WM microstructural properties, specifically diffusion anisotropy (i.e., direction dependence)^86^ were represented by the GFA value^44^, which was estimated with the formula: (standard deviation of the ODF)/(root mean square of the ODF)^44^. The GFA value is the most widely accepted measure in DSI and q-ball imaging and has a high linear correlation with the fractional anisotropy value derived based on the diffusion tensor model in DTI^109^. Based on the study investigating the plasticity in the motor network of stroke patients^110^, the observed decreased GFA values might link to disruption or loss of the axonal structures, whereas a GFA increase might be related to axonal sprouting or myelin growth. Despite this speculation, GFA (or any diffusion anisotropy measure such as fractional anisotropy) is only an indirect measures of axonal and myelin properties.

### Whole-brain tract-based automatic analysis

For the whole-brain tract analysis, the TBAA method was used to enable efficient tract-based analysis of the major fiber tracts over the entire brain^42^. Briefly, all the DSI datasets were first registered to create a study-specific template (SST), which was then registered to the DSI template (NTU-DSI-122)^111^. The DSI template (NTU-DSI-122) is a DSI data set averaged over 122 registered DSI datasets of healthy adults. A total of 76 tracts have been constructed by deterministic tractography using open software (DSI Studio: https://dsi-studio.labsolver.org). We then transformed the predefined 76 fiber tract bundles from the DSI template into the individual DSI data sets by transforming the sampling coordinates from the DSI template to the SST and then to the individual DSI data sets. GFA values were sampled on the tract coordinates of the 76 tract bundles. In this study, the mean GFA value was calculated from the GFA profile sampled along with each tract bundle in each participant. Supplementary Fig. 3 depicts the sample GFA profiles which record the GFA variability of the sampled tracts.

### Statistical analysis

Data analyses on group comparisons of demographic data and mean GFA values were conducted by using SAS 9.4 version (SAS Institute, Cary, NC). The alpha value was preselected at 0.05. The demographic data were compared by independent sample *t*-test (Table 1). The mean GFA values of the whole-brain white matter tracts between ASD and TDC were compared using the general linear model with a linear and square term of age, signal dropout counts, and SNR as covariates (Supplementary Table 2). To control for multiple tests in 76 tracts, a false discovery rate (FDR, *q*) correction was set at *q* < 0.05.

### Canonical correlation analysis

We implemented CCA, in steps similar to those previously reported^41,112,113^, to identify modes that relate WM microstructural property of 76 tracts with dysregulation levels and cognitive measures across the whole cohort (ASD + TDC).

In detail, the WM property matrix *N*_*1*_ (76 × 164) was normalized and demeaned per the procedure reported in Smith et al.^41^ (https://fsl.fmrib.ox.ac.uk/analysis/ HCP-CCA/hcp_cca.m), resulting in a matrix *N*_*2*_ for subsequent analyses. The potential confounding effects of head motion and age (i.e., signal dropout counts, SNR, and linear and square terms of age) were regressed from *N*_*2*_ to yield *N*_*3*_. A principal component analysis was then implemented using the FSLNets toolbox^114^ to reduce the dimensionality of the deconfounded WM property matrix (*N*_*3*_) to twenty eigenvectors (explaining 80.63% of the total variance in the *N*_*3*_ matrix). The data were reduced to this resolution to balance between maintaining the information within the datasets and avoiding overfitting the CCA. Notably, we acknowledge that no consensus exists for component number selection^115^.

Four subject measures were chosen to be included in the CCA: diagnosis, dysregulation levels, diagnosis by dysregulation interaction, and FIQ. Four CCA modes, which consisted of weighted linear combinations of orthogonalized non-imaging measures and WM property eigenvectors patterns, were generated. Each mode *m* stands for the maximum latent co-variation between these two combinations of the brain and behavioral variates in decreasing rank order. The vectors *U*_*m*_ and *V*_*m*_ denoted each participant’ weights for subject measures and WM property matrices within mode *m*, respectively:

The vector *U*_*m*_ represented the level of which each participant is (positively or negatively) correlated to population variation in subject measures within the mode *m*. The vector *V*_*m*_ is the extent to which each participant is correlated to population variation in WM property within the mode *m*. *R*_*m*_ represented the population covariation in the mode *m*, and was calculated by the correlation between *U*_*m*_ and *V*_*m*_. Familywise error (FWE)-corrected alpha < 0.05 was estimated via 10,000 per-mutations of the rows of one matrix relative to the other, to determine the statistical significance of each CCA mode.

We next assessed which WM tracts were most powerfully expressed by population variations in the original sets of WM property captured by CCA mode *m*. First, to obtain the relative weight (and directional signs) of each tract associated with the WM property patterns within mode *m*, we correlated *V*_*m*_ with the original WM property estimates in *N*_*3*_, resulting in a vector *A*_*Fm*_. In line with that has been previously done^41,112,113^, the top 25% highest absolute values within *A*_*Fm*_ defined WM tracts, which were most strongly covaried, either positively or negatively, with subject behavioral measures.

The other sensitivity analysis for CCA based on the primary sample (59 ASD and 62 TDC) was implemented using the ‘leave-five-out per diagnosis group’ approach, with 1,000 permutations. The average *p* value of the first CCA mode from these permutations was calculated.

## Supplementary information

Supplementary file1.
