# Supplementary material for: Association of self-regulation with white matter correlates in boys with and without autism spectrum disorder
Source: Sci Rep. 2020 Aug 14;10:13811. doi: 10.1038/s41598-020-70836-y (PMC7429820; doi:10.1038/s41598-020-70836-y)

**Association of self-regulation with white matter correlates in boys with and without autism spectrum disorder**

Hsing-Chang Ni^1,2^, Hsian-Yuan Lin^3,4,5^, Wen-Yih Isaac Tseng^6,7*^, Susan Shur-Fen Gau^2,3,6*^

**Supplementary Tables: Page 1-12**

**Supplementary Figure: Page 13-15**

**Supplementary Table 1.** Co-occurring psychiatric disorders and methylphenidate use in individuals with autism spectrum disorder (ASD)

|  | ASD (n=59) |
| --- | --- |
| **Co-occurring psychiatric disorders** | |
| ASD only | 31 |
| Co-occurring ADHD only | 16 |
| Co-occurring ADHD and ODD | 10 |
| Co-occurring ADHD and tic disorder | 1 |
| Co-occurring ADHD and learning disorder | 1 |
| **Methylphenidate** | 17 |

ADHD, attention-deficit/hyperactivity disorder; ODD, oppositional defiant disorder

**Supplement Table 2.** Comparison of the mean GFA values of the 76 white matter tracts between ASD and TDC

| Fiber tract Mean (SD) | ASD  (n=59)  GFA value | TDC  (n=62)  GFA value | Uncorrected *P*-value | Corrected *q*-value^a^ |
| --- | --- | --- | --- | --- |
| L_AF | 0.416 (0.038) | 0.403 (0.029) | .754 | 0.975 |
| R_AR | 0.404 (0.035) | 0.392 (0.026) | .771 | 0.975 |
| L_cingulum of main body component | 0.428 (0.054) | 0.412 (0.046) | .772 | 0.975 |
| R_cingulum of main body component | 0.447 (0.058) | 0.433 (0.048) | .997 | 0.997 |
| L_cingulum of hippocampal component | 0.312 (0.035) | 0.296 (0.033) | .760 | 0.975 |
| R_cingulum of hippocampal component | 0.375 (0.032) | 0.363 (0.036) | .852 | 0.975 |
| L_frontal aslant tract | 0.451 (0.040) | 0.442 (0.029) | .359 | 0.951 |
| R_frontal aslant tract | 0.361 (0.033) | 0.348 (0.026) | .827 | 0.975 |
| L_fornix | 0.347 (0.032) | 0.347 (0.031) | .805 | 0.975 |
| R_fornix | 0.370 (0.024) | 0.365 (0.023) | .924 | 0.975 |
| L_IFOF | 0.443 (0.034) | 0.433 (0.031) | .339 | 0.951 |
| R_IFOF | 0.406 (0.031) | 0.400 (0.027) | .040 | 0.551 |
| L_ILF | 0.370 (0.043) | 0.365 (0.041) | .346 | 0.951 |
| R_ILF | 0.380 (0.041) | 0.372 (0.039) | .053 | 0.551 |
| L_perpendicular fasciculus | 0.296 (0.030) | 0.294 (0.033) | .202 | 0.903 |
| R_perpendicular fasciculus | 0.315 (0.030) | 0.308 (0.032) | .873 | 0.975 |
| L_SLFI | 0.487 (0.028) | 0.468 (0.031) | .504 | 0.975 |
| R_SLFI | 0.512 (0.033) | 0.495 (0.029) | .450 | 0.975 |
| L_SLFII | 0.403 (0.040) | 0.391 (0.034) | .248 | 0.942 |
| R_SLFII | 0.405 (0.036) | 0.390 (0.036) | .779 | 0.975 |
| L_SLFIII | 0.421 (0.043) | 0.407 (0.038) | .533 | 0.975 |
| R_SLFIII | 0.368 (0.043) | 0.356 (0.042) | .710 | 0.975 |
| L_stria terminalis | 0.331 (0.025) | 0.333 (0.031) | .758 | 0.975 |
| R_stria terminalis | 0.328 (0.025) | 0.327 (0.026) | .908 | 0.975 |
| L_UF | 0.310 (0.031) | 0.308 (0.029) | .011 | 0.551 |
| R_UF | 0.308 (0.028) | 0.307 (0.025) | .058 | 0.551 |
| L_CST of hand | 0.530 (0.025) | 0.511 (0.025) | .104 | 0.659 |
| R_CST of hand | 0.591 (0.028) | 0.574 (0.026) | .470 | 0.975 |
| L_CST of trunk | 0.615 (0.030) | 0.598 (0.026) | .132 | 0.717 |
| R_CST of trunk | 0.563 (0.023) | 0.547 (0.024) | .099 | 0.659 |
| L_CST of mouth | 0.600 (0.028) | 0.586 (0.029) | .792 | 0.975 |
| R_CST of mouth | 0.578 (0.032) | 0.564 (0.032) | .756 | 0.975 |
| L_CST of toe | 0.591 (0.023) | 0.584 (0.027) | .492 | 0.975 |
| R_CST of toe | 0.588 (0.026) | 0.577 (0.024) | .408 | 0.956 |
| L_CST of geniculate fibers | 0.506 (0.027) | 0.486 (0.027) | .227 | 0.936 |
| R_CST of geniculate fibers | 0.527 (0.028) | 0.509 (0.029) | .415 | 0.956 |
| L_FS of OFC | 0.346 (0.039) | 0.332 (0.036) | .383 | 0.951 |
| R_FS of OFC | 0.350 (0.039) | 0.339 (0.031) | .055 | 0.551 |
| L_FS of VLPFC | 0.309 (0.032) | 0.310 (0.029) | .023 | 0.551 |
| R_FS of VLPFC | 0.310 (0.031) | 0.305 (0.029) | .500 | 0.975 |
| L_FS of DLPFC | 0.447 (0.036) | 0.438 (0.033) | .376 | 0.951 |
| R_FS of DLPFC | 0.455 (0.035) | 0.446 (0.030) | .301 | 0.951 |
| L_FS of precentral gyrus | 0.431 (0.034) | 0.419 (0.029) | .561 | 0.975 |
| R_FS of precentral gyrus | 0.409 (0.037) | 0.403 (0.035) | .143 | 0.725 |
| L_Medial lemniscus | 0.556 (0.029) | 0.530 (0.030) | .096 | 0.659 |
| R_Medial lemniscus | 0.608 (0.035) | 0.581 (0.034) | .194 | 0.903 |
| L_TR of VLPFC | 0.392 (0.026) | 0.385 (0.027) | .234 | 0.936 |
| R_TR of VLPFC | 0.394 (0.029) | 0.389 (0.028) | .048 | 0.551 |
| L_TR of DLPFC | 0.492 (0.029) | 0.479 (0.026) | .919 | 0.975 |
| R_TR of DLPFC | 0.485 (0.031) | 0.475 (0.026) | .468 | 0.975 |
| L_TR of precentral gyrus | 0.477 (0.030) | 0.466 (0.031) | .308 | 0.951 |
| R_TR of precentral gyrus | 0.485 (0.029) | 0.477 (0.027) | .899 | 0.975 |
| L_TR of postcentral gyrus | 0.458 (0.029) | 0.448 (0.025) | .671 | 0.975 |
| R_TR of postcentral gyrus | 0.481 (0.030) | 0.471 (0.027) | .870 | 0.975 |
| L_TR of auditory nerve | 0.275 (0.022) | 0.267 (0.022) | .824 | 0.975 |
| R_TR of auditory nerve | 0.285 (0.022) | 0.276 (0.022) | .709 | 0.975 |
| L_TR of optic radiation | 0.477 (0.031) | 0.466 (0.029) | .827 | 0.975 |
| R_TR of optic radiation | 0.464 (0.028) | 0.452 (0.029) | .895 | 0.975 |
| Anterior commissure | 0.198 (0.020) | 0.207 (0.028) | .629 | 0.975 |
| Posterior commissure | 0.385 (0.020) | 0.371 (0.022) | .124 | 0.717 |
| CC of genu | 0.429 (0.046) | 0.435 (0.045) | .054 | 0.551 |
| CC of DLPFC | 0.469 (0.042) | 0.468 (0.035) | .320 | 0.951 |
| CC of VLPFC | 0.470 (0.040) | 0.462 (0.037) | .373 | 0.951 |
| CC of SMA | 0.560 (0.042) | 0.543 (0.035) | .983 | 0.996 |
| CC of precentral gyrus | 0.528 (0.033) | 0.511 (0.035) | .937 | 0.976 |
| CC of paracentral lobule | 0.585 (0.043) | 0.569 (0.037) | .954 | 0.980 |
| CC of inferior parietal lobule | 0.537 (0.033) | 0.522 (0.035) | .388 | 0.951 |
| CC of postcentral gyrus | 0.543 (0.039) | 0.528 (0.037) | .879 | 0.975 |
| CC of superior parietal lobule | 0.489 (0.044) | 0.480 (0.038) | .652 | 0.975 |
| CC of superior temporal gyrus | 0.504 (0.031) | 0.496 (0.031) | .280 | 0.951 |
| CC of middle temporal gyrus | 0.450 (0.033) | 0.442 (0.031) | .650 | 0.975 |
| CC of temporal pole | 0.343 (0.023) | 0.333 (0.021) | .735 | 0.975 |
| CC of hippocampus | 0.379 (0.036) | 0.365 (0.037) | .847 | 0.975 |
| CC of amygdala | 0.449 (0.029) | 0.438 (0.030) | .604 | 0.975 |
| CC of precuneus | 0.465 (0.062) | 0.428 (0.061) | .090 | 0.659 |
| CC of splenium | 0.459 (0.045) | 0.451 (0.046) | .772 | 0.975 |

^a^Corrected *q*-value with false discovery rate correction for multiple comparisons

Abbreviations: AF: arcuate fasciculus; CC: corpus callosum; CST: corticospinal tract; FS: frontal-striatum; IFOF: inferior frontal occipital fasciculus; ILF: inferior longitudinal fasciculus; L: left; OFC: orbitofrontal cortex; R: right; SLF: superior longitudinal fasciculus; SMA: supplementary motor area; TR: thalamic radiation; UF: uncinate fasciculus; DLPFC: dorsolateral prefrontal cortex; VLPFC: ventrolateral prefrontal cortex.

**Supplementary Table 3.** The significant canonical correlation analysis (CCA) mode (*p*<0.05, family-wise error corrected) of the primary analysis.

| **CCA mode** | One |
| --- | --- |
| ***df_1_*** | 80 |
| ***df_2_*** | 385.07 |
| ***F*** | 1.53 |
| ***r*** | 0.587 |
| ***Wilk’s lambda*** | 0.337 |
| ***Familywise error corrected p*** | 0.005 |

**Supplementary Table 4.** Canonical correlation analysis (CCA) mode GFA weight and associated tracts

| **Tracts** | **CCA tract GFA modulation** |
| --- | --- |
| CC of genu | -0.310 |
| L_ILF | -0.288 |
| L_UF | -0.278 |
| L_CST of mouth | 0.244 |
| L_TR of auditory nerve | 0.260 |
| L_cingulum of hippocampal component | 0.261 |
| L_TR of precentral gyrus | 0.268 |
| Posterior commissure | 0.269 |
| L_cingulum of main body component | 0.272 |
| R_frontal aslant tract | 0.273 |
| R_TR of postcentral gyrus | 0.275 |
| L_CST of toe | 0.290 |
| R_CST of geniculate fibers | 0.309 |
| R_CST of trunk | 0.345 |
| L_CST of geniculate fibers | 0.353 |
| L_CST of trunk | 0.356 |
| R_CST of toe | 0.359 |
| L_Medial lemniscus | 0.368 |
| L_CST of hand | 0.398 |

Abbreviations: CC: corpus callosum; CST: corticospinal tract; ILF: inferior longitudinal fasciculus; L: left; R: right; SMA: supplementary motor area (SMA); TR: thalamic radiation; UF: uncinate fasciculus.

**Supplementary Table 5.** Demographic and clinical features of the originally recruited sample

| Mean (SD) | **ASD**  (n=87) | **TDC**  (n=77) | Statistics *P* value |
| --- | --- | --- | --- |
| **Age** (in years) | 13.6 (2.3) | 11.3 (2.4) | 0.001 |
| **Handedness**, right (%) | 81 (93.1) | 76 (98.7) | 0.122 |
| **Full-scale IQ** | 102.5 (17.2) | 109.8 (10.9) | 0.001 |
| Verbal IQ | 103.0 (16.7) | 110.7 (10.7) | 0.001 |
| Performance IQ | 101.7 (17.5) | 107.8 (13.1) | 0.013 |
| **Impaired self-regulation** | 210.3 (43.7) | 151.6 (30.5) | < 0.001 |
| **Autism Diagnostic interview-Revised**^a^ | |  |  |
| Social | 10.2 (4.8) | - |  |
| Communication | 5.5 (2.6) | - |  |
| Repetitive and  stereotyped behaviors | 8.7 (3.8) | - |  |
| **Head motion and Image Quality** | |  |  |
| Signal-to-noise ratio | 28.3 (2.9) | 26.7 (3.1) | 0.001 |
| Signal dropout counts | 11.6 (14.6) | 10.8 (12.1) | 0.715 |
|  |  |  |  |

^a^Based on the Current Behavior Algorithms

Abbreviations: ASD, autism spectrum disorder; TDC, typically developing controls; IQ, intelligence quotient; SD, standard deviation

**Supplementary Table 6.** The significant canonical correlation analysis (CCA) mode (*p*<0.05, family-wise error corrected) based on the originally recruited sample (ASD87/TDC77)

| **CCA mode** | One |
| --- | --- |
| ***df_1_*** | 120 |
| ***df_2_*** | 519.40 |
| ***F*** | 1.28 |
| ***r*** | 0.609 |
| ***Wilk’s lambda*** | 0.3559 |
| ***Familywise error corrected p*** | 0.0318 |

**Supplementary Table 7.** Canonical correlation analysis (CCA) mode GFA weight and associated tracts based on the originally recruited sample (ASD87/TDC77)

| **Tracts** | **CCA tract GFA modulation** |
| --- | --- |
| L_fornix | -0.305 |
| L_stria terminalis | -0.261 |
| R_fornix | -0.255 |
| R_UF | -0.191 |
| L_UF | -0.186 |
| L_FS of OFC | -0.184 |
| L_SLF I | 0.295 |
| R_TR of postcentral gyrus | 0.289 |
| L_TR of auditory nerve | 0.284 |
| L_TR of postcentral gyrus | 0.264 |
| L_perpendicular fasciculus | 0.262 |
| L_TR of precentral gyrus | 0.260 |
| R_CST of toe | 0.237 |
| L_cingulum of main body component | 0.221 |
| L_CST of toe | 0.211 |
| L_cingulum of hippocampal component | 0.198 |
| R_TR of auditory nerve | 0.196 |
| L_CST of hand | 0.195 |
| CC of postcentral gyrus | 0.189 |

Abbreviations: CC: corpus callosum; CST: corticospinal tract; FS: frontal-striatum; L: left; OFC: orbitofrontal cortex; R: right; SLF: superior longitudinal fasciculus; TR: thalamic radiation; UF: uncinate fasciculus.

**Supplementary Table 8.** Similar and different associations between dysregulation and GFA values between autism spectrum disorder (ASD) and typically developing controls (TDC) based on the originally recruited sample (ASD87/TDC77)

| **Pattern** | **Tract** | **Connected ROIs** | **Connected ROIs** | **System** |
| --- | --- | --- | --- | --- |
| **Similar association** | Not significant | | | |
| **Different association** | | | | |
| *a) The lower GFA values with the worse regulation in ASD/the better regulation in TDC* | | | | |
|  | Left fornix | L_mammillary body | L_hippocampus | Memory and emotion |
|  | Right fornix | R_mammilary body | R_hippocampus | Memory and emotion |
|  | Left Striatum terminalis | L_septal nuclei | L_amygdala | Emotion processing |
|  | Left UF | L_orbitofrontal gyrus | L_superior temporal pole | Emotion regulation |
|  | Right UF | R_orbitofrontal gyrus | R_superior temporal pole | Emotion regulation |
|  | Left FS of OFC | L_striatum (putamen+caudate) | L_orbitofrontal gyrus | Emotion regulation |
| *b) The higher GFA values with the worse regulation in ASD/the better regulation in TDC* | | | | |
|  | Left cingulum of main body component | L_cingulate gyrus  (anterior + middle) | L_cingulate gyrus  (posterior part) | Attention and cognitive control |
|  | Left cingulum of hippocampal component | L_cingulate gyrus posterior part | L_hippocampus | Memory |
|  | Left perpendicular fasciculus | L_angular gyrus | L_temporal-parietal gyrus | Attention, eye movement control and motion perception |
|  | Left SLF I | L_superior frontal gyrus | L_precuneus | Attention and motor control |
|  | Left CST of hand | Brain stem | L_primary motor cortex of hand component | Motor |
|  | Left CST of toe | Brain stem | L_primary motor cortex of toe component | Motor |
|  | Right CST of toe | Brain stem | R_primary motor cortex of toe component | Motor |
|  | Left TR of precentral gyrus | L_thalamus | L_precentral gyrus | Sensorimotor integration |
|  | Left TR of postcentral gyrus | L_thalamus | L_postcentral gyrus | Sensory processing |
|  | Right TR of postcentral gyrus | R_thalamus | R_postcentral gyrus | Sensory processing |
|  | Left TR of auditory nerve | L_thalamus | L_Heschl’s gyrus | Sensory processing |
|  | Right TR of auditory nerve | R_thalamus | R_Heschl’s gyrus | Sensory processing |
|  | CC of postcentral gyrus | L_postcentral gurys | R_postcentral gyrus | Sensory processing |

Abbreviations: CC: corpus callosum; CST: corticospinal tract; FS: frontal-striatum; L: left; OFC: orbitofrontal cortex; R: right; SLF: superior longitudinal fasciculus; TR: thalamic radiation; UF: uncinate fasciculus.

**Supplementary Figure 1.** Canonical correlation analysis (CCA) mode in the originally recruited cohort. (A) The CCA analysis identified a single significant (FWE-corrected *p*=0.032) mode of associations between white matter microstructural property and the behavioral variables of interest. The strength and direction of the variance explained by the CCA mode are indicated in the figure by the vertical position and font size. (B) Higher dysregulation levels in ASD, while lower dysregulation levels in TDC, were negatively correlated with white matter property of a set of 6 tracts including the bilateral fornix (purple), left striatum terminalis (SF, blue), bilateral uncinate fasciculus (UF, green), and frontostriatal tracts of the orbitofrontal cortex (OFC, red). (C) Higher dysregulation levels in ASD, while lower dysregulation levels in TDC, were positively correlated with white matter property of a set of 13 tracts including the main body (M, blue) and hippocampal (H, peach) components of the left cingulum, left perpendicular fasciculus (PF, yellow), left superior longitudinal fasciculus I (SLF, emerald)**,** the hand part of the left corticospinal tract (CST, crimson), toe part of bilateral corticospinal tracts (cyan), left thalamic radiation (TR) linking to the precentral gyrus (pink), bilateral thalamic radiation linking to the postcentral gyrus (yellow), bilateral thalamic radiation linking to the auditory nerve (royal blue), and postcentral part of the corpus callosum (CC, orange)**.** R = right; L = left; A = anterior; GFA = generalized fractional anisotropy.


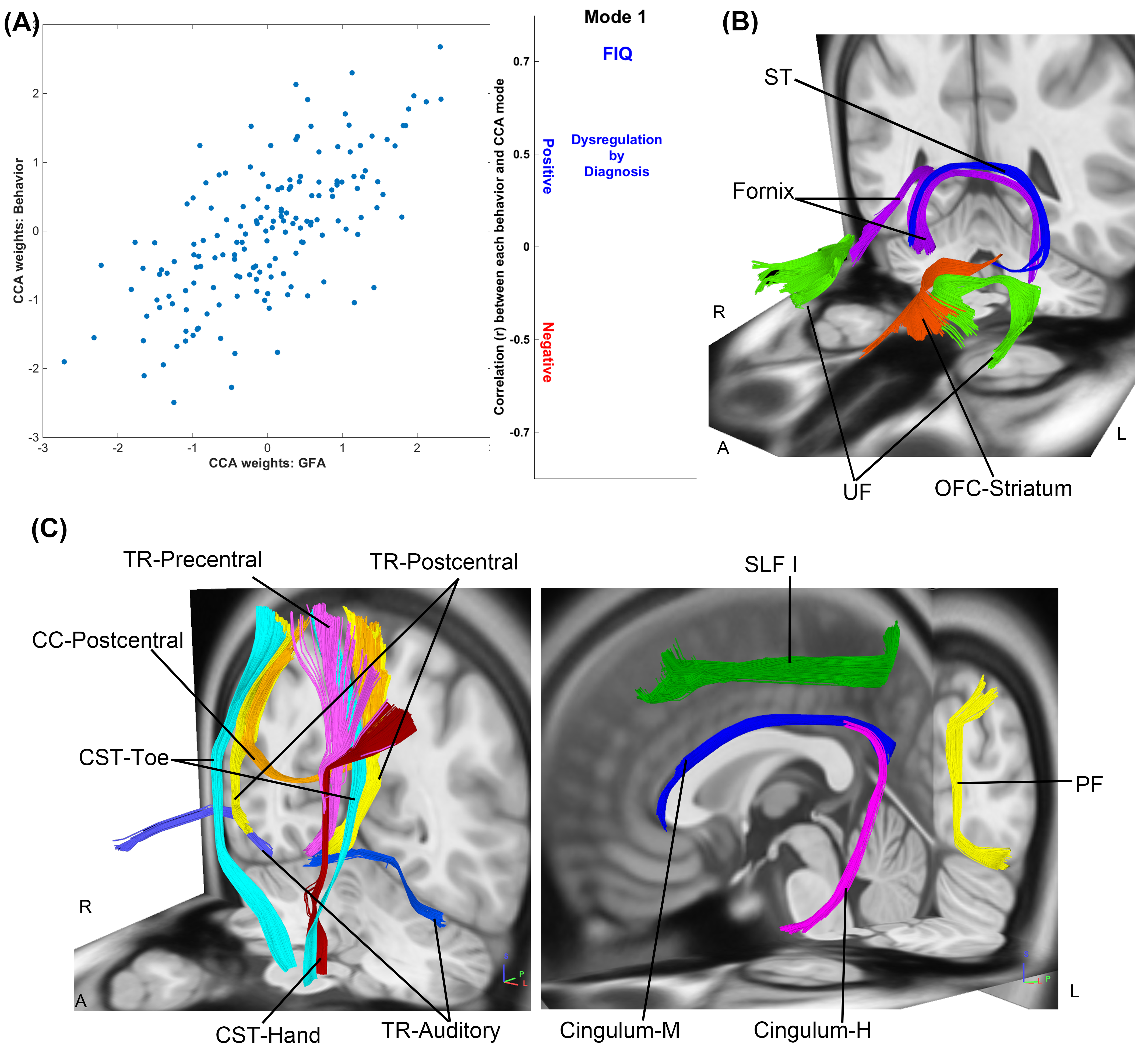


**Supplementary Figure 2.** The exact delineation of the reconstructed tracts (taking left ILF, left UF and CC of genu as examples) for 2 participants with ASD and 2 TDCs (randomly selected for the purpose of display of individual’s tractogram). Examples of individual tractograms from another 6 participants from each group could be downloaded at https://drive.google.com/drive/folders/169FgE-KdtsjGXocVC5-LWKxc3ICUKd33?usp=sharing.

**
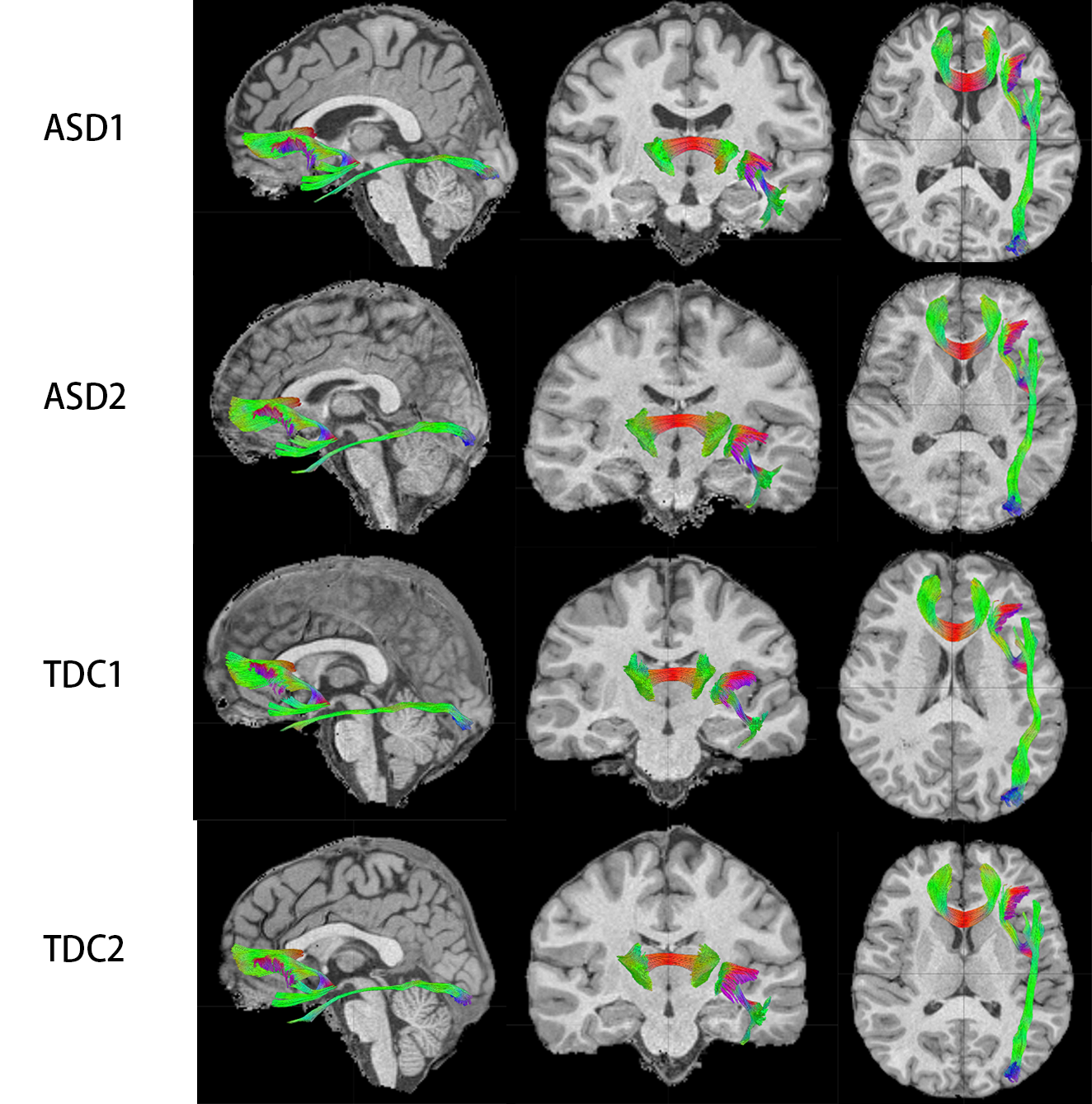
**

**Supplementary Figure 3.** The GFA profiles which record the GFA variability of the left ILF, left UF and CC of genu for the ASD and TDC groups (20 participants for each group, randomly selected for the display purpose)

ASD_L_ILF TDC_L_ILF


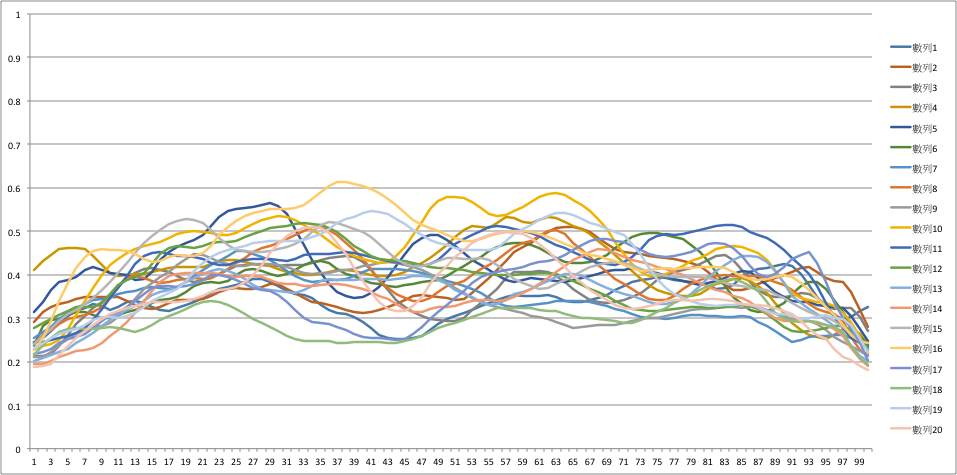

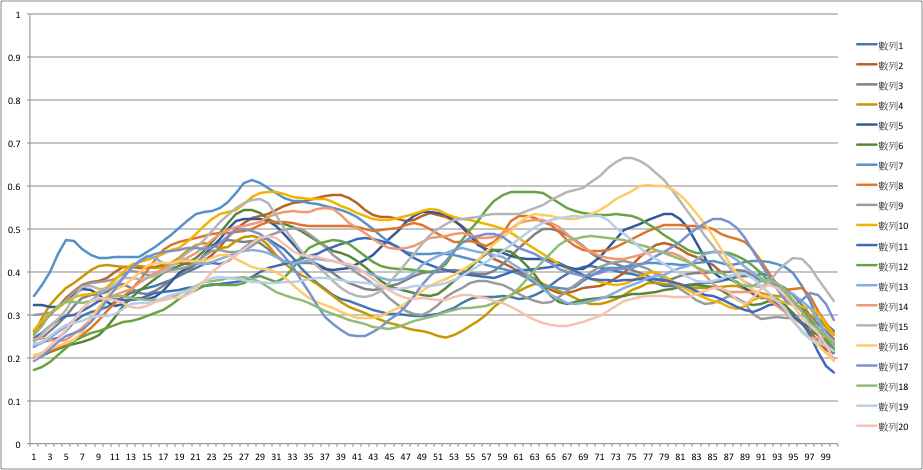


ASD_L_UF TDC_L_UF


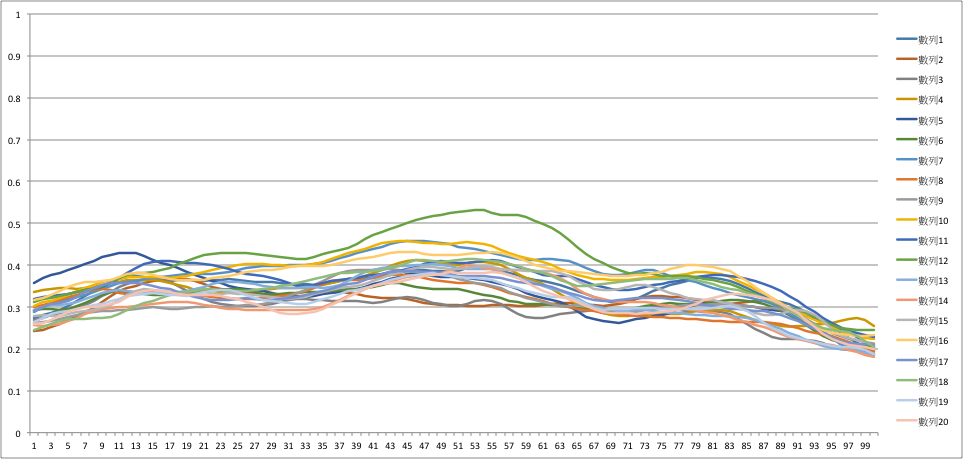

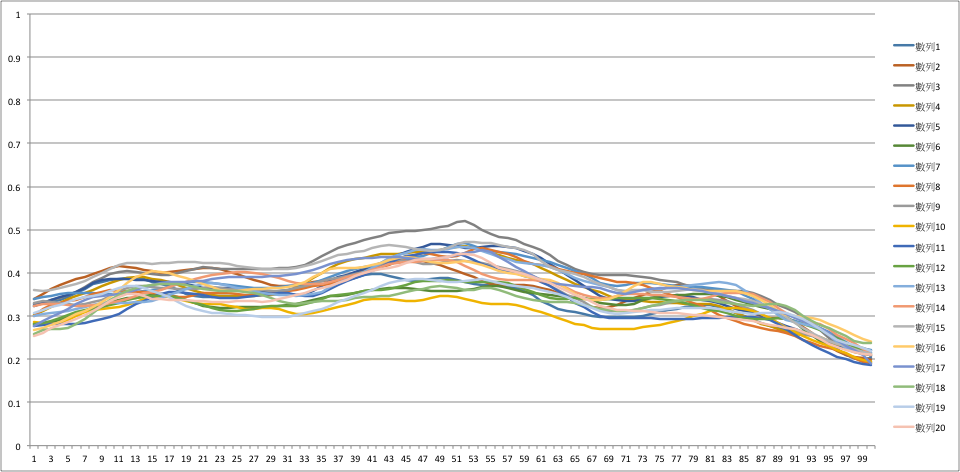


ASD_CC_Genu TDC_CC_Genu


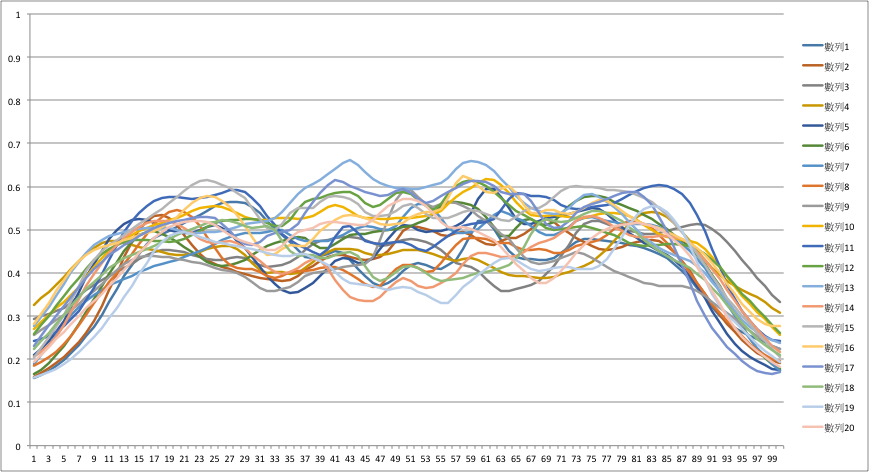

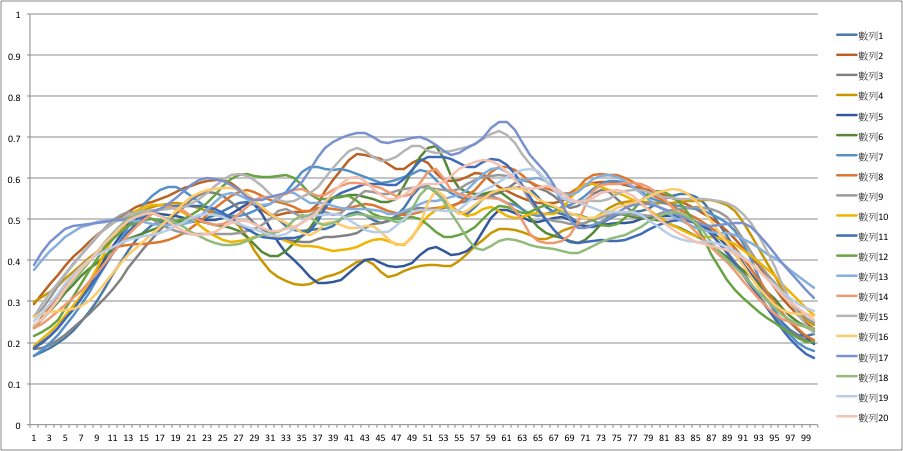

Supplement: Supplementary file 1 — Supplementary file1. [file 41598_2020_70836_MOESM1_ESM.docx]
